# Concerns Expressed by Chinese Social Media Users During the COVID-19 Pandemic: Content Analysis of Sina Weibo Microblogging Data

**DOI:** 10.2196/22152

**Published:** 2020-11-26

**Authors:** Junze Wang, Ying Zhou, Wei Zhang, Richard Evans, Chengyan Zhu

**Affiliations:** 1 College of Public Administration Huazhong University of Science and Technology Wuhan China; 2 Non-traditional Security Center Huazhong University of Science and Technology Wuhan China; 3 School of Medicine and Health Management Huazhong University of Science and Technology Wuhan China; 4 College of Engineering, Design and Physical Sciences Brunel University London London United Kingdom; 5 School of Political Science and Public Administration Wuhan University Wuhan China

**Keywords:** coronavirus, COVID-19, social media, public health, Sina Weibo, public opinion, citizen concerns

## Abstract

**Background:**

The COVID-19 pandemic has created a global health crisis that is affecting economies and societies worldwide. During times of uncertainty and unexpected change, people have turned to social media platforms as communication tools and primary information sources. Platforms such as Twitter and Sina Weibo have allowed communities to share discussion and emotional support; they also play important roles for individuals, governments, and organizations in exchanging information and expressing opinions. However, research that studies the main concerns expressed by social media users during the pandemic is limited.

**Objective:**

The aim of this study was to examine the main concerns raised and discussed by citizens on Sina Weibo, the largest social media platform in China, during the COVID-19 pandemic.

**Methods:**

We used a web crawler tool and a set of predefined search terms (*New Coronavirus Pneumonia*, *New Coronavirus*, and *COVID-19*) to investigate concerns raised by Sina Weibo users. Textual information and metadata (number of likes, comments, retweets, publishing time, and publishing location) of microblog posts published between December 1, 2019, and July 32, 2020, were collected. After segmenting the words of the collected text, we used a topic modeling technique, latent Dirichlet allocation (LDA), to identify the most common topics posted by users. We analyzed the emotional tendencies of the topics, calculated the proportional distribution of the topics, performed user behavior analysis on the topics using data collected from the number of likes, comments, and retweets, and studied the changes in user concerns and differences in participation between citizens living in different regions of mainland China.

**Results:**

Based on the 203,191 eligible microblog posts collected, we identified 17 topics and grouped them into 8 themes. These topics were pandemic statistics, domestic epidemic, epidemics in other countries worldwide, COVID-19 treatments, medical resources, economic shock, quarantine and investigation, patients’ outcry for help, work and production resumption, psychological influence, joint prevention and control, material donation, epidemics in neighboring countries, vaccine development, fueling and saluting antiepidemic action, detection, and study resumption. The mean sentiment was positive for 11 topics and negative for 6 topics. The topic with the highest mean of retweets was domestic epidemic, while the topic with the highest mean of likes was quarantine and investigation.

**Conclusions:**

Concerns expressed by social media users are highly correlated with the evolution of the global pandemic. During the COVID-19 pandemic, social media has provided a platform for Chinese government departments and organizations to better understand public concerns and demands. Similarly, social media has provided channels to disseminate information about epidemic prevention and has influenced public attitudes and behaviors. Government departments, especially those related to health, can create appropriate policies in a timely manner through monitoring social media platforms to guide public opinion and behavior during epidemics.

## Introduction

On June 29, 2020, the World Health Organization (WHO) marked the six-month anniversary of the COVID-19 outbreak [1]. The first case of the unknown pneumonia strain, now known as COVID-19 and caused by SARS-CoV-2, was reported in Wuhan City, Hubei Province, on December 31, 2019, and has subsequently made a profound impact worldwide. After clusters of pneumonia cases were reported in Wuhan in early January 2020, laboratory analyses were conducted that led to the epidemic being identified as a new coronavirus, officially named SARS-CoV-2 by the WHO. By July 3, 2020, 83,545 confirmed cases were reported in Mainland China [2]. After the outbreak of COVID-19 in China, the disease quickly spread globally. At the end of August 18, 2020, a total of 214 countries and regions had reported confirmed cases of COVID-19 worldwide, with the total number of cases exceeding 21 million and the total number of deaths worldwide exceeding 760,000 [3]. The global average mortality rate of COVID-19 is approximately 0.3%-1.5%; however, the mortality rates of COVID-19 in countries such as the United States, Brazil, and Mexico are much higher. With the worldwide spread of COVID-19, the epidemic has gradually attracted widespread attention and discussion on social media platforms. This trend was particularly evident on Sina Weibo, one of the largest social media platforms in China.

Extant studies have demonstrated that timely understanding of public attitudes and demands plays an important role in responding to public crises [4,5]. Most social media platforms possess media-oriented features that are crucial to mediating information dissemination. Citizens can receive the latest fact-checked information provided by governments on social media in a timely manner, while governments can use the publicly available information released by citizens to better understand public attitudes, concerns, and demands [6-8]. Sina Weibo, with over 500 million users by May 2020, provides a variety of communication mechanisms for citizen interaction, allowing the Chinese public to share information and exchange opinions [9-11]. By analyzing the concerns raised by citizens about COVID-19 in microblog posts shared on Sina Weibo, governments can better understand public attitudes and demands [12,13] and clarify existing challenges faced by government departments and organizations when dealing with the pandemic. This research provides important insights and implications for policy makers, especially those working in public health departments. The results offer deeper understanding of public perception and highlight shortcomings in practice to better meet public needs.

A large body of literature has examined the role of social media in analyzing public behavior, attitudes, and responses during times of public crisis. Researchers have mainly focused on the platform functionality, user behavior characteristics, and use of social media during crises. As most platforms are freely available in the public domain [14], they have become widely adopted methods for citizens to stay connected, discuss concerns and opinions, and escape the monotony of lockdown during the COVID-19 pandemic [15]. Researchers agree that social media has become an important medium for information dissemination during the pandemic [16] and is playing a unique role in information sharing [17] and health care discussion [18]. Some scholars have explored user behavior and connection networks on social media platforms and believe that personality characteristics affect users’ behavior [19], while networks comprise both positive and negative connections [20,21]. The unique position and roles of social media in response to public crises have also attracted major attention from researchers. They concur that social media platforms play important roles in crisis management, especially in terms of providing citizens with timely information [22] and reducing citizens’ anxiety and fear [23]. Based on existing research, in this paper, we collect data from the leading Chinese microblogging platform, Sina Weibo, to analyze the main concerns expressed by citizens during the COVID-19 pandemic.

### Methodology

#### Data Collection

Sina Weibo is the leading microblogging platform in China; it enables users to send and receive short character-limited posts and retrieve textual content by searching for specified keywords over a defined date range. Using this functionality, we collected shared microblog posts related to COVID-19 during the time period of December 1, 2019, to July 31, 2020. The Octopus web crawler tool was used to search for predefined keywords, including *New Coronavirus Pneumonia*, *Coronavirus*, *New Coronavirus*, and *COVID-19*. In addition to the textual content collected, we sourced the metadata for each microblogged post, including number of likes, number of comments, number of retweets, publishing time, and publishing location. The location information in the metadata refers to the registered address of the Sina Weibo account associated with a microblog post. To obtain these data, we used the advanced search functionality of Sina Weibo.

#### Data Preprocessing

In the Chinese language, there is no obvious separation between words. Therefore, for the purpose of completing the latent Dirichlet allocation (LDA) processing tasks, it was necessary to add obvious separators between words. We performed Chinese segmentation on the textual content collected during data preprocessing. A widely used Chinese word segmentation tool, ictclas, was employed to divide microblog posts into groups of words separated by spaces. For example, the microblog post “JD announced the donation of 1 million medical masks and 60,000 pieces of medical supplies to Wuhan City in batches” was segmented into “JD / announced / the / donation of / 1 million medical masks / and / 60,000 pieces of / medical supplies / to / Wuhan City / in batches”. In addition, we removed stop words [24] from the texts, similar to removing stop words such as “an” and “the” in English text analysis. Chinese stop words mainly fall into the following two categories: widely used vocabulary, such as me, you, some, and every day, and auxiliary words for mood, adverbs, prepositions, conjunctions, and other words that have no meaning by themselves, such as in, yes, so, and then [25]. In addition, punctuation and characters such as emojis were removed.

#### Data Analysis

We applied topic modeling by specifying the number of topics required by the LDA to separate the set of microblog posts into defined clusters [26]. LDA can be used to identify the most common topics in microblog posts shared on Sina Weibo. Topic modeling is an unsupervised machine learning technique that can identify clusters in a collection of documents (microblog posts in our case). In this study, we used the LDA algorithm from the LDA4j package. LDA4j is implemented in the Java language of the LDA algorithm, and the project can be downloaded for free from GitHub [27].

LDA is a widely used topic modeling algorithm [28]. According to the LDA model, a document (ie, the text of a microblog post) is a collection of vocabularies and may include multiple topics. The goal of the LDA model is to speculate on the distribution of topics based on a given document [29]. With LDA modeling, we can map the given documents to a fixed set of topics and capture the representative words for each topic. Then, the natural clusters in the microblog post dataset can be established.

To determine the appropriate number of LDA topics, we used the coherence score to draw judgments; this method has been proposed to be useful for selecting a suitable number of LDA topics [30]. Through continuous adjustment of the number of topics, we found that when the number of topics was 17, the consistency score reached its optimal value [31]. Therefore, we set the LDA model to separate the set of microblog posts into 17 clusters; the text sets are provided in Multimedia Appendix 1.

Subsequently, we conducted manual analysis and selected representative and high-proportion keywords from the top 30 keywords of each topic. Next, consensus was reached on the 17 topics and related keywords. Lastly, we used these keywords to classify the microblog posts; we also obtained the number of microblog posts under each topic and the proportion of each topic in all related microblogs. Examples of microblog posts for each topic are provided in Multimedia Appendix 2.

An example microblog is as follows:

The sudden outbreak of new coronal pneumonia has had an impact on the country’s economic operation. It has brought a negative GDP growth and an increasingly complicated international and domestic environment. However, under the strong hedging of the counter-cyclical adjustment policy, the resumption of production has advanced rapidly, while the main economic indicators show a rebound in March, and the decline rate narrowed significantly.

This microblog post can be categorized into two different topics: work and production resumption and economic shock.

We also performed other analyses of the collected data, such as sentiment analysis. The sentiment scores varied between –1.0 and 1.0, with –1.0 being the most negative text and 1.0 being the most positive text. In addition, we calculated the interaction rate of users for each topic by analyzing the average number of retweets, likes, and comments per topic. Finally, using the publishing time and location, we analyzed changes in user concerns based on the time period and differences in user engagement in discussions related to COVID-19 based on the Chinese region.

## Results

Using the web crawler tool and predefined search terms, we obtained a total of 203,191 microblog posts from the Sina Weibo platform that were shared between December 1, 2019, and July 31, 2020.

### Microblog Analysis

#### Topics

According to the results obtained from the LDA and the keywords involved in each topic, we were able to group the topics into 8 themes: (1) patient admission; (2) treatment and research; (3) treatment resources; (4) fighting the epidemic together; (5) work to restore order; (6) prevention and control measures of COVID-19; (7) domestic and overseas pandemic situation; and (8) impact of COVID-19. The corresponding terms for each topic are shown in Table 1; these keywords were also used as the criteria for topic classification.

**Table 1:** Topics covered in microblog posts and the representative terms corresponding to each topic.

Theme	Topics	Terms corresponding to each topic
Patient admission	Patients’ outcry for help	*help*, *attention*, *diffusion*, *receiving*
Treatment and research	COVID-19 treatments	*discharged from the hospital*, *cure*, *treatment*, *rehabilitation*, *Chinese Medicine*
	Vaccine development	*vaccines*, *prevention*, *drugs*, *clinical trials*
Treatment resources	Medical resources	*medical treatment*, *doctor*, *medical care*, *nurse*, *mobile cabin*, *ward*
Fighting the epidemic together	Material donation	*mask*, *materials*, *donations*
	Fueling and saluting antiepidemic action	*work with one heart*, *saluting*, *unity is strength*, *responsibility*, *persevere*, *fight*
Work to restore order	Work and production resumption	*resumption of work*, *resumption of production*, *employment*, *operation*
	Study resumption	*return to school*, *student*, *school*, *high school*, *college entrance examination*
Prevention and control measures of COVID-19	Quarantine and investigation	*quarantine*, *contact*, *fever*, *14 days*, *investigation*
	Joint prevention and control	*headquarters*, *work leading group*, *joint defense*, *joint control*
	Detection	*nucleic acid*, *positive*, *negative*
Domestic and overseas pandemic situation	Domestic epidemic	*Wuhan*, *Hubei*, *prison*, *Zhejiang*, *Shandong*
	Epidemics in neighboring countries	*Japan*, *South Korea*, *Tokyo*, *Russia*
	Epidemics in other countries worldwide	*America*, *Trump*, *Britain*, *Italy*, *India*, *Brazil*, *France*
	Epidemic statistics	*confirmed cases*, *new cases*, *cumulative cases*, *suspected cases*
Impact of COVID-19	Economic shock	*economy*, *influence*, *market*, *shock*
	Psychological influence	*hope*, *worry*, *fear*, *terrible*

#### Theme 1: Patient Admission

The topic contained in this theme is patients’ outcry for help, which relates to patients who have or may have contracted COVID-19 and their treatment situation. The cries for help of patients who are awaiting treatment have attracted widespread attention, while the subsequent reception and treatment of these patients has also attracted significant attention.

#### Theme 2: Treatment and Research

This theme contains two topics. The first topic is COVID-19 treatments. In this topic, the recovery of patients with COVID-19 received widespread attention, such as the number of people discharged from hospital and the number of people who recovered. At the same time, treatment methods have attracted widespread public attention, such as treatments using Chinese medicine. The second topic in this theme is vaccine development. In this topic, research progress in relation to vaccines and their clinical trials has attracted much comment. Meanwhile, there has been widespread debate about whether vaccines can achieve the goal of preventing the virus.

#### Theme 3: Treatment Resources

The topic in this theme is medical resources. This topic focuses on core aspects of medical resources; medical staff and hospital wards, the allocation and integration of medical resources, and the establishment of temporary hospitals such as mobile cabin hospitals are all of general concern.

#### Theme 4: Fighting the Epidemic Together

This theme includes two topics. The first topic is material donation. In the early stages of fighting the epidemic in mainland China, masks and other antiepidemic materials were extremely scarce. This issue aroused widespread concern, and the donation of various antiepidemic materials became an active topic. The second topic is fueling and saluting antiepidemic action. This topic includes two aspects; the first aspect is the confidence and determination to beat the pandemic. Keywords such as *fighting* and *defeat* represent the general attitude of Sina Weibo users towards the fight against COVID-19. The second aspect, cooperation in the fight against the pandemic, is often discussed; key phrases such as *work with one heart* and *unity is strength* were frequently mentioned.

#### Theme 5: Work to Restore Order

As the spread of COVID-19 in mainland China is gradually being controlled, the restoration of production and resumption of normal ways of living is becoming an active topic. This topic includes two subtopics. The first subtopic relates to work and production resumption. The progress and arrangements of the resumption of work and production have aroused widespread concern and discussion. This work, which is aimed at restoring normal production and operational order, has received widespread support from the Chinese public. The second topic is study resumption, which mainly involves two aspects: first, the time when students of all ages will return to school, and second, the time for the national college entrance examination.

#### Theme 6: Prevention and Control Measures of COVID-19

This theme includes three topics. The first topic is quarantine and investigation, which includes two aspects: the investigation of symptoms, such as cough and fever, and quarantine periods. In particular, the 14-day quarantine period has become standard.

The second topic is joint prevention and control. Taking measures of joint prevention and control and establishing a headquarters are important actions taken by the Chinese government in response to the epidemic. This action is also key for the Chinese government to contain the epidemic in the short term. Correspondingly, it has also been widely recognized and is of great concern to the public.

The last topic is detection. The main content related to this topic is nucleic acid detection, which is an important way to establish which patients have COVID-10 or have been infected with SARS-CoV-2. The topic of detection has aroused widespread discussion.

#### Theme 7: Domestic and Overseas Pandemic Situation

Four topics are included in this theme. The first topic is domestic epidemic. On the one hand, because Hubei and Wuhan are the main battlefields in the fight against the epidemic in China, the epidemic situation in these regions has received a significant amount of attention. On the other hand, the spread of the epidemic in China is also of great concern to the public. Epidemics in neighboring countries is the second topic. The peak of this topic appeared in mid-to-late February 2020, when the epidemic began to spread from China to neighboring countries, such as Japan, South Korea, and Russia. This has aroused heated discussion among Sina Weibo users.

The third topic is epidemics in other countries worldwide. At the time when this topic appeared, the epidemic had spread globally; therefore, the topic included many countries and regions. The United States, as the country hit hardest by the epidemic, has attracted widespread attention from Sina Weibo microbloggers. In particular, US President Donald Trump’s statement that if COVID-19 deaths in the United States could be controlled to less than 100,000, “we all together have done a very good job” [32] has been a subject of heated discussion. At the same time, other countries with serious numbers of cases, such as Italy, India, Brazil, and France, have caused extensive debates. The fourth topic is epidemic statistics. The epidemic data for COVID-19, such as the numbers of confirmed cases, new cases, and suspected cases, has received much attention and discussion; public attention to and discussion of the epidemic data have continued throughout all stages of the evolution of the epidemic. The epidemic statistics can be divided into two parts: domestic epidemic data and international epidemic data. Domestic epidemic data refers to case data released by the National Health Commission. For example, by the end of July 31, 2020, a total of 78,989 cases had been cured in China, and a total of 84,337 confirmed cases had been reported. International epidemic data is published by governments worldwide [33]. For example, by the end of July 31, 2020, the number of daily diagnosed cases globally exceeded 289,000, totaling 17.4 million [34]. The United States alone added more than 70,000 diagnosed cases in one day [35].

#### Theme 8: Impact of COVID-19

This theme has two topics. The first is economic shock. This topic mainly refers to the negative impact of the epidemic on economies and markets worldwide. The second topic is psychological influence. On the one hand, the outbreak of the epidemic has delivered a substantial psychological blow to the public, and emotions such as worry and fear have spread among citizens. On the other hand, the public also hopes and believes that the fight against the epidemic will be successful, and they remain in an optimistic mood.

### Results of the Proportional Analysis of the Main Topics

To understand the specific degrees of concern for the various topics identified, we established the number and proportion of each topic by counting the number of microblog posts per topic. For example, the proportion of each topic is the number of microblog posts shared about the topic divided by the total number of microblogs (N=203,191). The results of our analysis are shown in Table 2.

As shown in Table 2, statistics relating to the pandemic can be viewed as a metric to ascertain citizen understanding of the current situation during the epidemic. The topic of pandemic statistics has received significant attention since the initial outbreak and is ranked first, with 26.8% of posts (54,513/203,191). The domestic epidemic situation has always been the focus of public attention, especially when the epidemic in mainland China had not been fully controlled. The number of microblog posts on this topic accounted for 18.1% of the posts (54,513/203,191), ranking it second. As COVID-19 spread worldwide, the number of microblog posts referring to the pandemic in other countries grew consistently; this topic is ranked third with a percentage of 13.9% (28,329/203,191).

The treatment of COVID-19 raised high expectations and received attention from the public for a long period, with a proportion of 11.1% (22,725/203,191). Medical resources are particularly important in the fight against the epidemic, and this topic accounts for 10.9% of posts (22,246/203,191). The great impact of COVID-19 on the economy is closely related to the interests of the public; therefore, the topic of economic shock also received a high degree of attention, ranked sixth with 10.1% of posts (20,699/203,191). As one of the most important measures for controlling and preventing the spread of COVID-19, the topic of detection and quarantine has received considerable attention at all stages of the pandemic since its outbreak, accounting for 9.12% of total posts (18,541/203,191). The topic of patients’ outcry for help also attracted widespread attention, accounting for 8.61% of total posts (17,513/203,191).

**Table 2:** Numbers of microblog posts on Sina Weibo related to each topic (N=203,191), n (%).

Rank	Topic	Microblog posts
1	Pandemic statistics	54,513 (26.8)
2	Domestic epidemic	36,936 (18.1)
3	Epidemics in other countries worldwide	28,329 (13.9)
4	COVID-19 treatments	22,725 (11.1)
5	Medical resources	22,246 (10.9)
6	Economic shock	20,699 (10.1)
7	Quarantine and investigation	18,541 (9.12)
8	Patients’ outcry for help	17,513 (8.61)
9	Work and production resumption	15,914 (7.83)
10	Psychological influence	14,485 (7.12)
11	Joint prevention and control	12,457 (6.13)
12	Material donation	12,434 (6.11)
13	Epidemics in neighboring countries	11,995 (5.90)
14	Vaccine development	9428 (4.64)
15	Fueling and saluting anti-epidemic action	9413 (4.63)
16	Detection	8939 (4.39)
17	Study resumption	6889 (3.39)

An important measure to restore economic order in China is work and production resumption, which is closely related to citizens’ lives. The percentage of microblog posts on this topic is 7.83% (15,914/203,191). The epidemic also greatly affected the public psychologically; therefore, the topic of psychological impact also received much attention, with a post percentage of 7.12% (14,485/203,191). Joint prevention and control and material donations have received a certain amount of attention. These two topics ranked 11th and 12th, with proportions of 6.13% (12,457/203,191) and 6.11% (12,434/203,191), respectively. The situation in neighboring countries also aroused comment from Sina Weibo users, although the duration was short-lived; accordingly, this topic ranks 13th, with 5.90% of posts (11,995/203,191).

The development of vaccines has also been discussed by the public, with related microblog posts accounting for 4.64% of posts (9428/203,191). The fight against COVID-19 is a national battle in which citizens fuel discussions on how to overcome the pandemic. The percentage of posts relating to this topic was 4.63% (9413/203,191). As an important means of identifying patients who are infected with COVID-19, detection has also received a certain degree of attention, accounting for 4.39% (8939/203,191). After the pandemic in Mainland China was controlled, study resumption was also mentioned; however, compared to other topics, the degree of concern in this area is relatively low, accounting for only 3.39% of total posts (6889/203,191).

### Results of Users’ Interaction and Sentiment Analysis

Based on the collected microblog metadata, including the numbers of retweets, comments, and likes, we were able to analyze the levels of interaction between users for each topic. At the same time, we performed a sentiment analysis to obtain the sentiment value for each topic. Table 3 shows the average numbers of retweets, comments, and likes for each topic, as well as their sentiment values. The calculation formula used to determine the sentiment value is (a – b)/(a – b), where a is the number of tweets expressing positive emotions and b is the number of tweets expressing negative emotions [36].

It should be noted that the data reported on in this article included microblogs posted by opinion leaders. The numbers of retweets, comments, and likes for these microblog posts were high. For example, for the post “Zhong Nanshan said that Lianhuaqingwen capsules were proved to be effective in the treatment of COVID-19”, the numbers of retweets, comments, and likes were 13,599, 19,022, and 344,780, respectively. Therefore, the average numbers of retweets, comments, and likes in this paper are high.

From Table 3, it can be seen that domestic epidemic, material donation, and medical resources ranked as the top three topics in terms of the number of retweets, demonstrating that Sina Weibo users have a strong desire to share this information. By analyzing the number of likes, the mean was found to be relatively high for the following topics: quarantine and investigation, fueling and saluting antiepidemic action, material donation, and COVID-19 treatments. According to the sentiment value for each topic, the numbers of topics with positive and negative sentiment values were 11 and 6, respectively. Furthermore, for most topics, the sentiment value was low, which indicates that the number of microblog posts with positive emotions is almost the same as the number of microblog posts with negative emotions. However, negative emotions were obvious, except for those related to pandemic statistics.

**Table 3:** Results of user interaction and sentiment analysis for each topic.

Rank	Topic	Retweets (mean)	Comments (mean)	Likes (mean)	Sentiment value
1	Work and production resumption	8	12	87	0.16
2	Epidemics in neighboring countries	29	22	199	–0.006
3	Coronavirus treatments	26	26	244	–0.05
4	Vaccine development	24	16	199	0.08
5	Pandemic statistics	15	12	178	–0.11
6	Economic shock	18	10	97	0.051
7	Material donation	35	17	245	0.058
8	Domestic epidemic	71	20	184	0.024
9	Medical resources	34	35	241	0.031
10	Quarantine and investigation	33	24	280	–0.049
11	Psychological influence	29	29	217	0.041
12	Joint prevention and control	15	13	236	0.146
13	Study resumption	7	10	129	0.083
14	Fueling and saluting antiepidemic action	19	13	248	0.073
15	Epidemics in other countries worldwide	11	10	118	–0.117
16	Patients’ outcry for help	22	18	224	–0.017
17	Detection	14	16	129	0.041

### Results of Spatiotemporal Analysis of Microblog Posts

**Figure 1:**
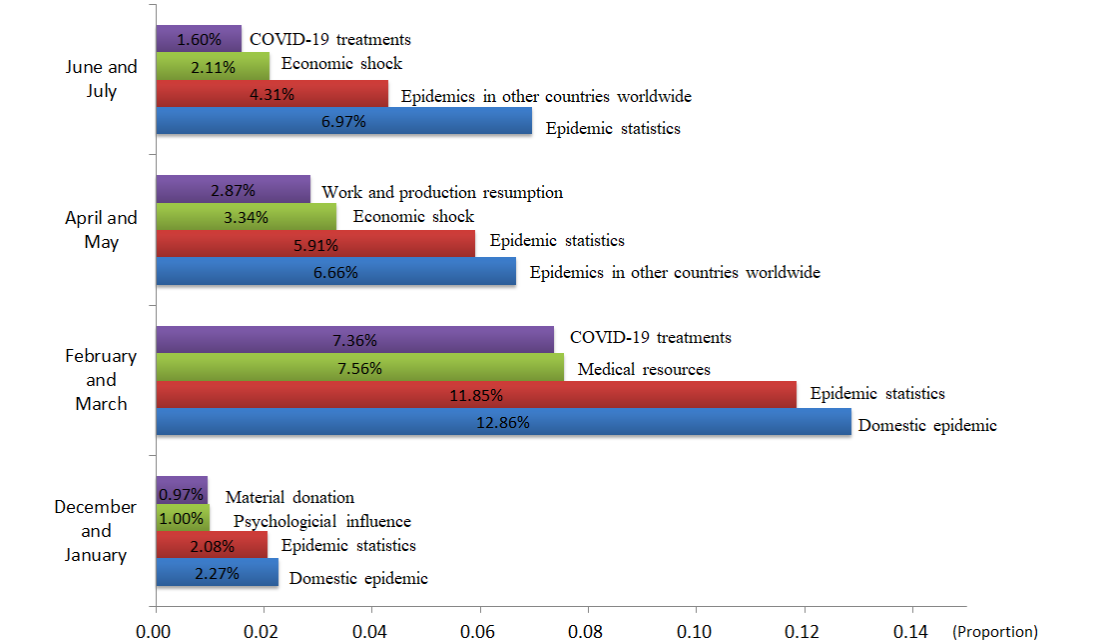
Changes in the proportions of posts about the main topics from January to May.

By using the publishing times of the microblog posts, we were able to analyze the change in users’ concerns during different time periods. Similarly, we used the publishing location to analyze the differences in user engagement in discussions relating to COVID-19 between different regions in China. Figure 1 illustrates how the proportions of topics changed over the time period. The figure includes the names and proportions of the top four topics with the highest levels of attention from December 2019 to July 2020. Considering the different months, the top four topics constantly changed. From December 2019 to July 2020, there were 9 different topics in the top four, including pandemic statistics, domestic epidemic, epidemics in other countries worldwide, COVID-19 treatments, medical resources, economic shock, work and production resumption, psychological influence, and material donation. In December 2019 and January 2020, four topics received a high level of attention: domestic epidemic, pandemic statistics, psychological influence, and material donation. In February and March 2020, the topics of domestic epidemic and pandemic statistics received more attention than before; the numbers of microblog posts on these topics ranked first and second, respectively. Meanwhile, the degrees of attention paid to the topics of medical resources and COVID-19 treatments increased, and they entered the top four topics. In April and May 2020, resumption of work and production and economic shock became active topics of discussion. At the same time, the topics of pandemic statistics and epidemics in other countries worldwide retained a high degree of attention. In June and July 2020, the most popular topics changed little compared with previous months. Topics such as pandemic statistics, epidemics in other countries worldwide, and economic shock also retained a high degree of attention during this period.

**Figure 2:**
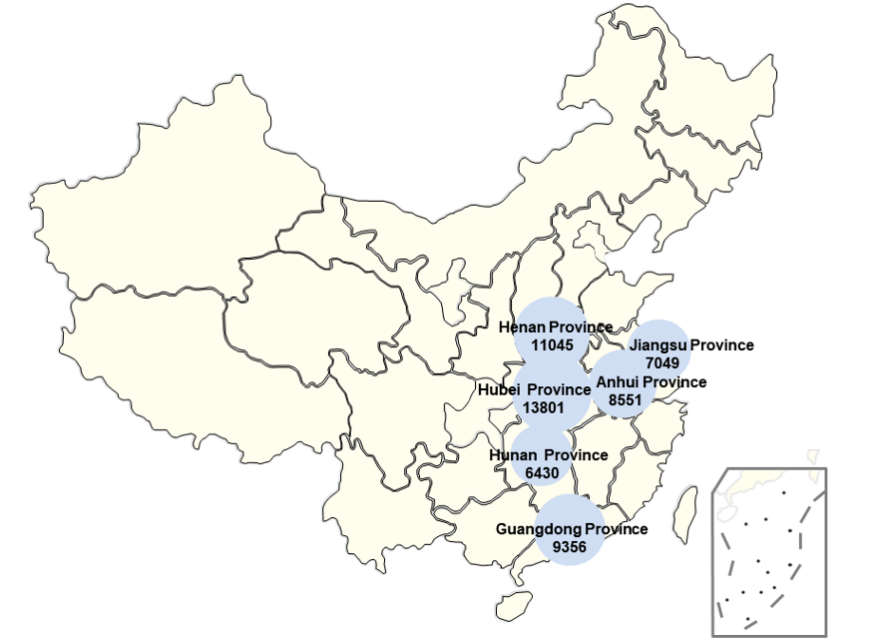
Numbers of microblog posts by Chinese province.

Figure 2 presents a geographical ranking of the number of microblog posts. The location information was obtained from the registration addresses of the Weibo accounts that posted the microblogs. Of the 203,191 microblog posts collected, 127,009 (62.5%) contained the publishing location. By analyzing the publishing location, we identified the six regions with the largest numbers of microblog posts. It can be seen from Figure 2 that a much higher number of posts originated from Hubei Province than from any of the other provinces. Henan Province and Guangdong Province were ranked second and third, respectively, with other provinces ranking below these.

## Discussion

### Main Findings

#### Sina Weibo Users Mainly Focus on the Latest News About COVID-19 Treatment and the Status of the Pandemic Domestically and Globally

Our study, conducted from December 1, 2019, to July 31, 2020, identified 8 themes with 17 topics. Among these topics, content related to COVID-19 treatment aroused great attention, particularly content related to patients’ outcry for help and emotions such as anxiety and panic. Due to the widespread negative emotions on Sina Weibo, misinformation and rumors can more readily influence public opinions, which negatively impacts antiepidemic actions. In addition, Sina Weibo users showed much interest in the progress of the treatment of COVID-19 and of vaccine development. At the same time, there has been considerable focus and discussion among a large number of Sina Weibo users on whether medical resources are sufficient and whether they can meet the treatment needs of patients.

Sina Weibo users also focused heavily on the real-time situation of the epidemic at home and abroad. In particular, many microblog posts contained real-time data on the development of the epidemic. Further, Sina Weibo users not only paid attention to the epidemic situation in the Chinese mainland but also greatly discussed the development and evolution of the epidemic in neighboring countries and the rest of the world. In addition, epidemic prevention and control measures, order restoration measures in the later stage of the epidemic, and the impact of the epidemic are included in the 17 topics that received attention from Sina Weibo users.

#### The Changes in the Number of Microblog Posts for the Various Topics in Different Time Periods Reflect the Process of the Occurrence, Development, and Demise of the COVID-19 Pandemic in China

Our findings show that December 2019 and January 2020 were the key periods of the response to the COVID-19 pandemic, with domestic epidemic and pandemic statistics receiving much attention during this period. At the same time, the sudden outbreak also affected the public psychologically. On the one hand, the public believes that the fight against the epidemic will eventually be won; on the other hand, emotions such as fear and worry are also spreading among the public.

February and March 2020 were critical periods for curbing the spread of the epidemic in mainland China [37]. During this period, pandemic statistics and the domestic epidemic were still causing high degrees of concern. At the same time, the surge in the number of patients caused the public to pay attention to COVID-19 treatments and medical resources. From April to May 2020, the pandemic in mainland China was basically under control; however, COVID-19 had begun to spread to other countries and regions. At this time, economic shock and work and production resumption became the most highly debated topics on Sina Weibo. Similarly, users paid great attention to the global spread of the pandemic and viewed real-time data.

From June to July 2020, the number of infections and deaths caused by COVID-19 worldwide continued to rise. The focus of Sina Weibo users shifted from China to the rest of the world, and the users began to pay greater attention to epidemics in other countries and the growing pandemic statistics.

#### The Location of Sina Weibo Users and Whether They Experienced Similar Events Are Closely Linked to Their Degree of Attention to Public Crises

According to the spatial distribution information collected on microblog posts, discussions related to COVID-19 differed among the various regions of China. The six regions with the highest degrees of participation were Hubei Province, Henan Province, Guangdong Province, Anhui Province, Jiangsu Province, and Hunan Province. Hubei Province is the epicenter of the COVID-19 epidemic in China, while Henan Province, Hunan Province, and Anhui Province are all adjacent to Hubei Province, with many citizens working in Hubei Province. Jiangsu Province, as an economically developed region, has a relatively high mobility rate of personnel. As a result, Sina Weibo users in these provinces were more likely to be concerned about the pandemic. Guangdong Province experienced the severe acute respiratory syndrome (SARS) epidemic in 2003, which is an important reason why more users in this region participated in discussions on the COVID-19 pandemic.

#### Concerns of Users of the Sina Weibo and Twitter Microblogging Platforms Show Similarities and Differences

A recent study reported the main topics related to COVID-19 that are of concern to Twitter users [36]. The authors analyzed 4 themes and 12 topics related to COVID-19 that Twitter users were concerned about from February 2 to March 15, 2020. They found that Twitter users were mainly concerned about the impact of COVID-19 on people and countries. For example, the number of deaths related to COVID-19 and the impact on citizens’ emotions and psychology were mentioned in many tweets. In addition, the economic impact of COVID-19 was widely discussed. In particular, Twitter users mentioned two main methods for reducing the spread of COVID-19: masks and quarantine. Compared with these findings, Sina Weibo posts shared some similarities. Topics including economic impact and psychological influence received high attention on both Sina Weibo and Twitter [36]. However, at the same time, the main concerns of Sina Weibo users demonstrated unique characteristics. First, Twitter users focused on the causes and effects of the epidemic, while Sina Weibo users paid greater attention to prevention, control, and treatment. Various epidemic prevention and control measures, such as isolation, detection, and joint prevention and control, have attracted widespread attention among Sina Weibo users. Similarly, Sina Weibo users are highly concerned about content related to COVID-19 treatments, such as patients’ outcry for help, medical resources, and treatment methods. In addition, citizens have continued to pay high degrees of attention to the development of the a vaccine against SARS-CoV-2. This may be due to the fact that the Chinese government has adopted a series of response measures and has made citizens aware of the severity and harmfulness of the pandemic; thus, Sina Weibo users are more concerned about health-related topics such as the prevention, control, and treatment of the virus.

Second, Sina Weibo users focus on the real-time status of the epidemic. This includes not only the domestic epidemic situation, but also the development of the epidemic abroad. At the same time, in the middle and late stages of the epidemic, work to restore order, such as work and production resumption and study resumption, have attracted widespread attention. The following two reasons may have led to the emergence of the above unique concerns. First, as Chinese citizens were the first nation to experience the COVID-19 pandemic, they are more sensitive to the development and changes of the epidemic than people in other countries, and second, as the country that has demonstrated the most effective epidemic prevention and control, China has established conditions to restore order [38].

#### Prompt Guidance of Negative Emotions on Sina Weibo Is of Paramount Importance

The results of our sentiment analysis show that of the 17 topics, the numbers of topics with positive sentiment values and negative sentiment values are 11 and 6, respectively; for most topics, users have no obvious positive or negative emotional tendencies. However, it should be noted that there are still some topics in which the emotional value of a certain aspect is significant. For example, topics such as pandemic statistics demonstrated negative emotional tendencies. The continuous accumulation and spread of negative emotions on Sina Weibo may trigger irrational behavior among citizens, causing users to be affected by rumors or extreme emotions [19], such as group panic and denial of government support; thus, timely guidance to address negative emotions is essential.

### Research Implications

With the outbreak and spread of COVID-19 worldwide, citizens have turned to social media channels, such as Sina Weibo, to share their opinions, seek clarity, and discuss topics related to the crisis. Previous studies have demonstrated that the analysis and control of public behavior and attitudes can effectively help governments cope in times of crisis [39]. As many countries start to experience a second wave of COVID-19 outbreaks, social media platforms can collect large amounts of information that reflect public behavior and attitudes. By analyzing this data, it is possible to identify the demands and behavioral characteristics of citizens [40].

In this paper, we collected data from Sina Weibo and analyzed it from the viewpoints of quantity, proportion, emotion, and space-time distribution. We identified the degrees of attention and the emotional tendencies of users toward various topics related to COVID-19, and we determined the time distribution for each topic and the spatial differences of users’ participation in topic discussions. The results obtained enable deeper understanding of the views and attitudes of the public towards COVID-19, which is the premise of and basis for the prevention and control of this novel disease.

### Strengths and Limitations

This paper is based on the existing theories of predecessors, combined with the timely topic of COVID-19. Through analysis of Sina Weibo microblogs, practical conclusions are drawn from the topics related to COVID-19 that are of concern to the public. At the same time, it is undeniable that this article also presents some limitations. First, the microblog posts collected in this study only include some posts related to COVID-19, not all of them. Therefore, the summary of the main concerns of Sina Weibo users is not comprehensive. Secondly, the majority of Sina Weibo users are young; therefore, the results of our analysis are more representative of younger citizens. Finally, although the number of Sina Weibo users is relatively large in China, we cannot draw a rushed conclusion that opinions expressed on the web represent public opinion in general.

### Conclusions

The COVID-19 outbreak has had a crippling impact on the world economy and has presented numerous challenges to how people live and travel. Topics related to COVID-19 have attracted widespread attention and discussion on social media platforms, such as Sina Weibo. By analyzing the microblog posts of COVID-19 topics, we obtained the opinions of citizens on topics related to the virus which can lead to the identification of solutions to solve societal and economic problems [41]. In light of the comparative analysis of the main topics of Sina Weibo and Twitter, the topics of posts by Sina Weibo users show more regional characteristics. Judging from the distribution of microblog posts, the proportion of each topic reveals obvious differences. According to the sentiment analysis results, citizens reveal a positive emotional attitude. However, some negative emotions remain among the public, which requires timely guidance from Chinese governments. By analyzing the time distribution of microblog posts, it was observed that the proportion of topics changed significantly during different periods, while the main concerns of users changed with the development of the pandemic. Similarly, by analyzing the publishing location of microblog posts, we identified that user participation in the discussion of topics related to COVID-19 revealed obvious regional differences.

## Appendix

Multimedia Appendix 1 Examples of microblogs for each topic.

Multimedia Appendix 2 Topics in Chinese.

## Abbreviations

LDA: latent Dirichlet allocation
SARS: severe acute respiratory syndrome
