# Supplementary material for: Concerns Expressed by Chinese Social Media Users During the COVID-19 Pandemic: Content Analysis of Sina Weibo Microblogging Data
Source: J Med Internet Res. 2020 Nov 26;22(11):e22152. doi: 10.2196/22152 (PMC7695542; doi:10.2196/22152)
Supplement: Multimedia Appendix 1 [file jmir_v22i11e22152_app1.docx]

**Table 1 Examples of microblogs for each topic**

| **Topic** | **Microblogs(example)** |
| --- | --- |
| Patients’ cry for help | On February 8, 2020, Li Lina struck gongs and cried on the balcony of the community, asking for help for her seriously ill mother. This detail during the epidemic have caused many concerns and disputes. |
| New Crown treatments | On the morning of the 11th, Mr. Chen, the last patient with new coronary pneumonia in Nanchong, Sichuan, was cured and discharged from Nanchong Central Hospital. So far, all 39 confirmed cases in Nanchong City have been discharged from the hospital, achieving "clearance". |
| Vaccine development | # The new crown vaccine may be available for emergency use in September# This is a great news, thanks to all the scientific research teams for their hard work. |
| Medical resources | The new crown pneumonia has not been over for so long. The army, supplies, and medical staff are still rushing to various places in Hubei. Mobile cabin hospitals  have been built one after another, and the country's manpower and material resources are concentrated on fighting pneumonia. |
| Material donation | I am an art candidate who has been in Wuhan for a month, and I am going to donate the masks I bought to Wuhan. The number is very small and I only hope to do my best. |
| Refueling and saluting towards anti-epidemic action | #All people are united in the fight against new coronary pneumonia# The community adopted measures such as closed management in advance, popularization of knowledge through the establishment of WeChat groups, and the self-tested body temperature report. Residents in the community are mostly retired university teachers and they have a strong sense of self-protection. "This is a two-way work, requiring the joint efforts of the community and residents." |
| Work and production resumption | Some people should not think that resumption of work is so terrible. The key is to take precautions. Shanghai has resumed work for 10 days, but no new cases of new coronary pneumonia have been confirmed in a day and a half. Reasonably arrange the resumption of work, strengthen management and prevent it before it happens, and use the wisdom of the Chinese to defeat the epidemic. Come on, everyone. |
| Study resumption | Under the new crown pneumonia epidemic, when can junior students return to school? Will the high school entrance examination this year be delayed? This is the education event that parents are most concerned about in this year. |
| [Quarantine](https://fanyi.so.com/?src=onebox#%20quarantine) and investigation | Zhao Jianping, director of the Department of Respiratory Medicine, emphasized that isolation is the first to fight the new coronavirus pneumonia. Once you find that you have symptoms, you must avoid going out, isolate yourself at home, and keep a distance of one meter when you are in contact with others. |
| Joint prevention and control | The State Council’s joint prevention and control mechanism established a liaison group to understand and coordinate the prevention and control of epidemic in Hubei Province and Wuhan City, and dispatched a supervision group to supervise and guide Heilongjiang Province to improve and strengthen prevention and control measures, and to check loopholes and fill shortcomings, especially Strengthen the prevention and control of nosocomial infections and go all out to do a good job in medical treatment. |
| Detection | Nucleic acid testing is one of the important criteria for diagnosing new coronary pneumonia. Recently, with the gradual advancement of resumption of work, resumption of production, and resumption of school, the central government has decided to carry out large-scale nucleic acid and antibody testing to improve normalized prevention and control. |
| Domestic epidemic | On February 21, the reporter learned from the Hubei Provincial Prison Administration that there were 271 confirmed cases of new coronary pneumonia in the Hubei Provincial Prison System, of which 230 were confirmed in Wuhan Women’s Prison in Hubei Province and 41 were confirmed in Shayang Hanjin Prison in Hubei Province, all of which were imported cases. |
| Epidemics in neighboring countries | On the 19th, the number of people diagnosed with the new crown virus in Japan and South Korea continued to increase. |
| Epidemics in other countries around the world | According to British media reports, a spokesperson for the World Health Organization said on the same day that the number of confirmed cases of new crown pneumonia in the United States has increased significantly and the United States may become the new epicenter of the global new crown epidemic. |
| Epidemic statistics | #Global epidemic trends# A total of over 250,000 cases of new coronary pneumonia outside Chinese mainland have been diagnosed. |
| Economic shock | The World Bank’s April issue of "Commodity Market Outlook" stated that the global economic shock caused by the pandemic has caused the prices of most commodities to fall, which is expected to lead to a sharp drop in prices in 2020. |
| Psychological influence | In 2020, a new crown epidemic that swept the world has changed our lives and broke everyone's work arrangements. Many people's work, life, and mental and physical health have been greatly affected. Because of the impact of the epidemic, I cannot go out to work, worrying that my life has no source; worrying that I and my family will be infected with new coronary pneumonia... |
