# Supplementary material for: Concerns Expressed by Chinese Social Media Users During the COVID-19 Pandemic: Content Analysis of Sina Weibo Microblogging Data
Source: J Med Internet Res. 2020 Nov 26;22(11):e22152. doi: 10.2196/22152 (PMC7695542; doi:10.2196/22152)
Supplement: Multimedia Appendix 2 [file jmir_v22i11e22152_app2.docx]

==============end==================

选择17个topic时，结果如下：

Sampling 1000 iterations with burn-in of 100 (B/S=20).

BBBBB|S||S||S||S||S||S||

S||S||S||S||S||S||S||S||S||S||S||S||S||S||S||S||S||S||S||S||S||S||S||S||S||S||S||S||S||S||S||S||S||S||S||S||S||S||

topic 0 :

复工=0.0441656556100572

企业=0.03492471667558364

复产=0.032363992037770926

疫情=0.0216423058469056

恢复=0.015600173287768831

服务=0.013818411031126259

防控=0.012523970251632382

保障=0.011565496078667406

生产=0.010181186544978843

支持=0.007594497609082285

有序=0.007205688850694741

期间=0.006920954714838841

政策=0.006751753652318057

工作=0.0061261540052087666

开放=0.005636872618277417

部门=0.005349570476890132

市场=0.0050964634276257454

助力=0.004976724064330835

相关=0.004858450245911739

项目=0.004753347703633853

生活=0.004745889358113591

发展=0.004596407965646914

出台=0.004573326485077456

就业=0.00437348523256868

我市=0.004351718241930502

确保=0.0041558900752026095

建设=0.004143867615374078

措施=0.004124337678918951

发生=0.004089306031708038

经营=0.003710779977481415

需求=0.003621725341933429

旅游=0.0035833206955278958

全市=0.003405345868434936

办理=0.0034025200311127166

防疫=0.0033132480330871393

群众=0.0032743404045532934

推进=0.0032290861789679107

影响=0.003091891804501909

提供=0.0030234197669421884

记者=0.0030160451273649434

单位=0.0028806567463008853

运营=0.0027783529611900043

行业=0.0026899707749369214

困难=0.002621800757599246

市民=0.0025984065217954525

景区=0.0025775872750714852

文化=0.002574890963912631

推动=0.0025403831036404853

餐饮=0.002518777158224966

有限公司=0.002516743595964065

物资=0.0025033077335865576

交通=0.0024105151313648177

组织=0.002379764042812103

集团=0.0023591696101115784

营业=0.002352452168583627

通知=0.0021957957833580906

出行=0.002174451788690339

减免=0.002168083660551951

税费=0.0021561204764151205

供应=0.0021278022220288764

业务=0.0021008323024438984

获悉=0.0020536174997297318

落实=0.001966145444379558

一手=0.0019631825745612566

条件=0.001948042918273118

环境=0.0019165083183155162

推出=0.0019106229197927108

公司=0.0018962116262935032

暂停=0.0018945944384406138

重点=0.0018838637314256865

加快=0.0018732305083600299

医保=0.0018531766372524474

监督=0.0018427998322640656

举措=0.001842685655651853

预约=0.0018339571016324594

发放=0.0018291638210349368

消费=0.0017910190379136835

切实=0.0017591558104696739

运输=0.0017539929292410847

员工=0.001725404773239201

高速=0.0017241924478942814

优惠政策=0.00171198739044332

专项=0.0017063308019127786

工程=0.0016888740778232588

联合=0.0016778185950601667

运行=0.0016395583267254305

临时=0.0016362447935506196

民生=0.0016174095588009083

全省=0.0016148914766669533

客运=0.0016018638932213916

系列=0.0015957011859829612

秩序=0.0015933392582760685

社会=0.0015907604391685456

形势=0.001583088118628576

出口=0.001574748550649755

江西=0.0015616236993973498

农村=0.0015591196040288075

基础=0.0014979916580841004

精准=0.0014895513993361637

产品=0.001487634131488175

topic 1 :

新冠=0.08898391448185174

肺炎=0.0822277746155756

日本=0.0388201019488202

感染=0.017090100868625648

韩国=0.014161740596827765

流行=0.008127245729534967

香港=0.007722543334186825

国籍=0.007600492942244041

报道=0.0072315620661333535

消息=0.006726954548235949

确诊=0.006563601006083704

公费=0.005572854991027328

取消=0.005026010029409212

东京=0.005007381700334745

邮轮=0.0049809663566871476

入境=0.004410036553169278

乘客=0.004333888681347416

政府=0.004242655004134756

今日=0.0042144002183396555

游客=0.0038734826015643935

举办=0.0038344671404920653

首例=0.0038107650847467576

俄罗斯=0.0037021657512103473

新加坡=0.0035818587333164035

费用=0.0033697258409175306

公主=0.0032649547959478966

劳动=0.0032503886909491147

钻石=0.003225796836847583

航班=0.003219117073812179

接受=0.003215020861370499

泰国=0.0031864455828212734

奥运会=0.003048214558761742

升级=0.0030291631861778085

采访=0.002853657293670942

措施=0.0028194879226037246

治疗=0.0028064930330486382

紧急=0.0026721847942086426

钻石公主号=0.0026532184511991845

中心=0.002586523312620359

回国=0.0025688709699442426

参加=0.0025296350269093295

上海=0.0025191408914328967

包括=0.0025118495096176086

发生=0.002505676119673505

总理=0.0024635155992037204

日本政府=0.002387379016446077

指定=0.0022921478336029194

航空=0.002275632119590944

集体=0.0022724434796926878

健康=0.002244341574456779

承担=0.002223354785312134

意味=0.002153651866048467

旅客=0.0021310544204610022

厚生=0.0021185619885627283

委员会=0.002118410133202258

陕西=0.002083091952893253

台湾=0.0020691389770104277

防疫=0.0020594873569819133

日本国=0.0020430679678113614

机场=0.0020091897872814594

农历=0.001959053484461596

首尔=0.001954418005259382

包机=0.0019240476683081939

谣言=0.0019100781171488997

强制=0.001909140868171417

认定=0.0018894629573091564

安倍晋三=0.0018850263057097378

10万=0.0018733308392421587

首次=0.0018586211624864078

禁止=0.001835094214194312

京津冀=0.0018251600522032603

海外=0.0017765335889618403

已有=0.001772249313360658

大邱=0.0017384921748803662

检疫=0.001716744162922003

功能=0.001706139449450548

增至=0.0016922555681589274

奥运=0.0016884672443496756

计划=0.0016609417339453396

感染者=0.0016543195906344851

明年=0.0015979958864921725

司机=0.0015968578227232705

三月=0.0015403606050698234

当天=0.0015091496614050928

通行=0.001503817557671071

基地=0.0014797695125063428

讨论=0.001465748945433973

星期一=0.0014513151845221824

超级=0.001419703766154037

安倍=0.0014174108652543734

人民币=0.0014128642585603478

男性=0.0013806306430328783

省内=0.001375174007477204

辟谣=0.0013650943519661622

隔离=0.0013516820445779257

大臣=0.0013459245213392431

乘坐=0.0013333322137999105

飞机=0.0013140791409698932

女性=0.0013106068025364895

确认=0.0012959657140827142

topic 2 :

患者=0.08943004305923068

新冠=0.05218961584736302

肺炎=0.04562938077983641

治疗=0.040655594044805454

出院=0.017077164911687183

重症=0.01503340590865697

治愈=0.014325994351744021

血浆=0.012815247520066493

救治=0.012096755938223543

逾期=0.008721638181499196

临床=0.008657305335618199

信用卡=0.008652689566630236

专家=0.00822218757797096

病人=0.008186602426752895

康复=0.007400020615191566

首例=0.007360348998916244

康复者=0.007018494402950215

病情=0.0069122399112235096

中医=0.006726793742024483

捐献=0.0064534611285945705

第一=0.005828521901780734

医院=0.005724661145887196

中药=0.005406025912241653

方案=0.0053572990931134

团队=0.0052466863759772645

银行=0.005237389164129248

中医药=0.005205054602853794

风险=0.005192962283383756

诊疗=0.005177919893092584

诊断=0.004457809786943496

时间=0.004423508188429651

告诉=0.004372328431305433

阶段=0.004349714312925214

人群=0.004340710133089416

机构=0.004194692040550894

心理=0.003857318508141344

人士=0.003838056776431001

缓解=0.00350837334025012

紧张=0.003396202202620632

成功=0.0033931136773007018

面临=0.0033175492201199747

标准=0.0033097785183185627

危重=0.003299689689195043

好消息=0.0032861063182895085

国家=0.003257756037411425

住院=0.003193317860547525

遗体=0.0030481550757076865

解剖=0.003009394967059294

金银潭医院=0.002991689956765841

CT=0.0029886984430545997

症状=0.002944969517911188

进展=0.002924134266863871

人民医院=0.0029001141601770032

管控=0.0028626849309942005

生物=0.00283886862572118

识别=0.0027319640473909

贷款=0.0027145094753249902

疾病=0.002709910506404672

接受=0.0026618579075362034

效果=0.002646658549928078

行业=0.002634273974529767

攀升=0.002597850149273351

工作=0.0025950674660312795

界面=0.0025907728171716713

年龄=0.0025756694662782565

新闻记者=0.002557409276249966

外部=0.002545817117619246

客户=0.0024821389383100417

股份=0.002467336663325442

冲击=0.002436520682801966

好转=0.0024131071079880454

痊愈=0.0023773286705869663

重压=0.0023393162568222177

抗体=0.0023134465569287295

身体=0.0022650324575072445

血液=0.002257525314666617

肺部=0.0022561382719471254

方法=0.0022310205094463404

催收=0.0021504911637326967

叫苦=0.002133262359735385

宽限=0.0021309687788745152

女士=0.0021227284771888548

张定宇=0.0020656055714811973

病理=0.002029134390959772

中西医=0.002018751899161152

用于=0.001961411095580731

下午=0.0019378662045888904

降低=0.0019073364643206077

献血=0.0018995094457955038

符合=0.0018263040276007003

呼吸=0.0018015335142384187

收治=0.0017826140582386182

第六=0.0017499038240049297

院长=0.0017364620224195753

过程=0.0017088124884312701

入院=0.0016913657267706782

ICU=0.0016777581943455448

恢复期=0.0015955595470679277

第五=0.0015843910043743029

卫健委=0.0015508036256463146

topic 3 :

病毒=0.0802591588669989

新冠=0.060093130271258476

传播=0.023581332256025555

疫苗=0.01700245031045629

研究=0.01685322420730635

钟南山=0.012986597904465725

发现=0.011848067118490659

预防=0.009136251867819009

药物=0.008743150396398171

传染=0.00844011578537388

流感=0.008322956222124225

研发=0.008124254743321974

疾病=0.006862052356037488

专家=0.006688128149235048

感染=0.006226568746894213

抗体=0.005941060830397998

科学=0.005201547634309261

传染病=0.00510792409885189

发表=0.005090184669361862

文章=0.00504302875297889

治疗=0.0043000531624881695

团队=0.004132890883349914

上市=0.0038552938622195953

感冒=0.0038238100864421425

临床试验=0.0037843207698473433

SARS=0.0036793418885664527

气溶胶=0.0036772563050194817

途径=0.0036674206588003648

院士=0.00366573151416519

免疫=0.0035671850286998937

控制=0.003440202871083824

分钟=0.003338364427509029

人类=0.003201271797983144

潜在=0.003130308712633114

获批=0.003048642368931884

氯喹=0.0029588009739515957

毒株=0.0028945513420903617

临床=0.0027907819165655184

科学家=0.0027631451166279285

实验=0.00268613570937148

科研=0.0026283184770688086

瑞德西韦=0.0025298003255367417

特效药=0.002504854449451759

科普=0.002435118955943259

研制=0.002390085565969789

作用=0.002360288683958836

工程院院士=0.0023024879393612236

成功=0.0022907199702487447

谣言=0.002263687041375378

分析=0.0022446565862455636

包括=0.0022128680687092993

免疫力=0.0021788583819101993

导致=0.002168710925479761

试验=0.002151332176438718

论文=0.002128928991750767

回应=0.0021257256143221003

证据=0.002111667335985199

出新=0.002077784706871092

呼吸道=0.0020761575259721087

人体=0.002046173098905994

COVID=0.002037922128835834

来源=0.0019948362533124607

显示=0.0019558704935973862

研究所=0.0019307363899135196

真相=0.0019295108827324246

热点=0.0018798771984395966

空气=0.0018774645847519393

基因=0.0018751874449142222

分离=0.0018586185705636675

易感=0.0018466475299628688

潜伏期=0.0018371685228132798

儿童=0.0018308368842102892

服用=0.0017904729697446197

症状=0.0017654087724719356

区分=0.0017568189963936961

防治=0.0017123873406008575

动物=0.0017079687763596795

飞沫=0.001702996406633208

中国疾控中心=0.0016827966653553028

实验室=0.0016609462024455668

判断=0.0016544839904052294

命名=0.001652198143263607

人群=0.0016384157219926413

源头=0.0016200175517968227

生物=0.001601220983037587

找到=0.001586014162595101

连花清瘟=0.0015679226614801975

传人=0.0015429793711280186

磷酸氯喹=0.0015222413562924048

辟谣=0.0015060813437576866

用于=0.0014853750144099536

呼吸=0.0014841429903212314

批准=0.0014786767302200391

疗效=0.0014579221745369701

群体=0.0014498178304899014

不可能=0.001431142467881972

最新=0.0013657832765108244

都是=0.0013624469656036149

阻断=0.0013501622529639979

快速=0.0013452019367749398

topic 4 :

病例=0.16385124541064428

确诊=0.14899364755745093

新增=0.0957986444451005

累计=0.05054436846962896

报告=0.033630770879036855

肺炎=0.030174168265275386

出院=0.02248388338780085

症状=0.01987431933042478

治愈=0.0178753912222327

境外输入=0.017632590838086395

感染者=0.01753522747368541

疑似=0.01564963635547966

死亡=0.013538599160809744

最新=0.012268133612314706

通报=0.010887114348606588

新冠=0.01001540915136368

连续=0.00874252370578752

情况=0.008454434317042774

观察=0.007500681170379606

新疆=0.006976410423309292

医学=0.006662994093010669

全省=0.006391288042232542

时至=0.005912519322500972

现有=0.005163357070777891

兵团=0.003917921029011932

自治区=0.0038131118027130554

北京=0.0037744631837816293

本土=0.0037347478947440753

宁夏=0.003560462749270816

解除=0.0033205894164715258

接触=0.003181391402433169

上海=0.0031228356341541437

当日=0.0030871201993216465

建设=0.0029812274313705182

输入=0.0029195801980123064

天津=0.002727501037633482

卫健委=0.0026321817353169713

公布=0.002423588230528838

全区=0.002411225669485238

生产=0.002369177000149932

重庆市=0.0023641630477307736

甘肃=0.0022371082930202828

俄罗斯=0.0022334519705816524

我市=0.00220531220249843

卫生=0.0021536301757324847

动态=0.0021125522492763723

关联=0.0020450141379560736

昨日=0.002030828640485401

大连=0.0020249400519489224

安徽省=0.0019347186110506395

境内=0.001904329047448319

发布=0.0018469165376915194

直辖市=0.0018442840426235827

辽宁=0.0018259845477328093

重庆=0.0018184730487793952

乌鲁木齐市=0.0017314832638902025

广州市=0.001655946613419708

全国=0.0016134808389245377

省区=0.0015418038954398596

新疆维吾尔=0.0014806987817769747

追踪=0.0014738575114507594

河南省=0.0014582031933942055

全市=0.0014279270422280141

重症=0.001383305064232295

广东=0.0013544756151623474

河南=0.0013361477254334106

广东省=0.00131429202727348

郑州市=0.0013139877126442537

重型=0.0012764572044769485

山西省=0.0012622265389448074

单日=0.0012102141077753301

海南=0.0011871417215127168

无疑=0.0011103986328863948

大连市=0.0011069524272188942

详情=0.0011034621930563445

甘肃省=0.0010099362890244998

河北=0.001005916256177131

乌鲁木齐=9.87982906381838E-4

播报=9.872486953818862E-4

口岸=9.153319441179281E-4

广州=9.127876168051576E-4

辽宁省=9.064918780413093E-4

信阳市=8.060336280596588E-4

河北省=7.823284152446873E-4

一例=7.67955316462867E-4

四川=7.591959688781317E-4

海南省=7.075863278883117E-4

江苏省=7.067854593510965E-4

安徽=6.962987529788941E-4

获悉=6.884022464941024E-4

吉林=6.796860924748361E-4

天津市=6.789126001445399E-4

疾控中心=6.527637365523551E-4

贵州省=6.291514805451497E-4

山西=6.243355399297854E-4

海河=6.237707999884125E-4

青海=6.124061592481404E-4

至此=5.886653709214905E-4

接受=5.601758567970892E-4

轻型=5.398926267820579E-4

topic 5 :

疫情=0.06155822073775927

经济=0.02263902746081419

影响=0.019032461897607163

全国=0.011608405910320876

全球=0.009355815900305715

下降=0.00892184924280019

公司=0.008862363727125521

市场=0.007254190297782768

我国=0.0072364013749846

增长=0.006756319047090878

持续=0.006616512567737045

发展=0.006486472241040565

形势=0.005777599884288825

一季度=0.005762939017168511

国内=0.005093779810554872

蔓延=0.00501513315153545

数据=0.0048448160204400525

月份=0.004509294895427748

冲击=0.004405161415568904

爆发=0.004285140512327156

带来=0.004107742089341044

增加=0.004040070422144552

首次=0.003905803470345665

世界=0.0038467836366481083

同比=0.003822560379880891

向好=0.0038040248255458682

控制=0.003779871235992483

地区=0.0037782564005201793

社会=0.003691437017175832

国家=0.0036905706508513496

情况=0.003495043041655324

美元=0.003465656099445627

面临=0.0033641528757045037

新冠=0.0033348808143234005

导致=0.003278873961288338

肺炎=0.003203459489726125

压力=0.0031237513265037747

员工=0.0030999414714142134

价格=0.0030921753033740485

中国=0.0030588175255640325

稳定=0.0029828088995740654

危机=0.002864558908802897

计划=0.00284705639066824

近期=0.002811375879665467

运行=0.0027717843577825824

减少=0.002748670375688175

收入=0.0027048368925028927

态势=0.0026991843199333804

严峻=0.0026841963623531005

消费=0.0026242248315898552

投资=0.00257978019775611

第一=0.002526807071514023

体系=0.002474957891813902

公共卫生=0.0024257410586843433

大幅=0.002405962085023043

国际=0.0023959751450399963

公布=0.002347714230457367

半年=0.0023394375209927305

面对=0.002224184537484308

财政=0.00222403882334574

去年=0.00221313614137335

挑战=0.002200792141490982

预计=0.002196957532309

销售=0.002190352137817117

发布=0.002173007018890159

加快=0.0021615140477017954

显示=0.002088273618970198

风险=0.0020634228800941413

能力=0.001986114226459546

未来=0.00198486025510211

损失=0.001983710844332786

产业=0.0019462131197235922

改革=0.001945075908707237

分析=0.0018852454955901158

办公=0.0018688553955205941

财经=0.0018289005784370396

变化=0.001824510365831087

生产=0.0018181636549308427

因素=0.0018169909016791206

高峰=0.001809639271645563

行业=0.0017820022012267464

水平=0.0017803317954570032

指数=0.0017530167475378682

汽车=0.0017441000534215191

程度=0.0017182336249064803

重启=0.0017065014057528196

需求=0.001701572364213915

降至=0.0016995308517894088

食品=0.0016905311956524653

规模=0.0016558446734784974

历史=0.0016461549778592787

城市=0.0016415261146905875

GDP=0.001608806148498101

季度=0.0016048008003035246

趋势=0.001604499110573461

总体=0.0015963430202079723

产品=0.0015906327831150516

制造=0.0015649897049438474

负责人=0.0015562538534769803

产业链=0.0015286494866369516

topic 6 :

中国=0.08463008027105824

疫情=0.042269850806610526

口罩=0.03622197593721453

新冠=0.029214336813956195

肺炎=0.022596195237320184

国家=0.019916379456670966

专家=0.016203523641011065

物资=0.0103053519990873

抗疫=0.01029357798871272

抗击=0.00966538220895411

捐赠=0.009079919913457413

国际=0.008898644057805138

希望=0.008721803580922312

世界=0.00805515022611914

社会=0.007172522684875676

张文宏=0.007141140063850198

建议=0.006212226845949433

支持=0.006183200015516203

教授=0.005132457256299051

互联网=0.004764425271248695

封城=0.004635592865727831

高度=0.004430345854752898

习近平=0.004375647797643621

组长=0.0041644184047069125

上海市=0.004146828606995819

人类=0.003761430648362397

回应=0.00368348491490056

警惕=0.0036775674977248124

方式=0.0036192469897380324

合作=0.003553110691565661

防护=0.0034729965117712173

发生=0.0034420135655673255

经验=0.0031743454824354846

越来越=0.0031500951122943825

关注=0.0031090059134937418

唯一=0.0031077379124107825

外交部=0.0030557874225365937

医用=0.0030527947678871243

疾病=0.003051460180307824

更好=0.003020183881260951

对此=0.002932187826667496

状态=0.0029000055018300096

感谢=0.002822426662112404

中方=0.0026877630587572384

应对=0.002661049249657122

提供=0.002604974711776123

空调=0.0025972878803654505

办法=0.002577233464270487

指出=0.0025051311194736773

重视=0.002480487784485808

记者=0.0024235252789216494

发展=0.0023849558280462425

规模=0.0023662240250407096

呼吁=0.002353827618838062

援助=0.002329242818864256

政治=0.0022590862263753043

手段=0.002246317516213042

海外=0.0022403058490334227

阶段=0.0022072674590724755

活下去=0.002140092446943815

全世界=0.0021323731071188827

发言人=0.0021058584922430965

聚会=0.0020980974140483557

各国=0.002078010052031876

放弃=0.0020117700883534556

白皮书=0.002011512697742211

方向=0.002004768711574002

成效=0.0020043719633873174

努力=0.0020005047911427175

目的=0.0019099386550167439

接受=0.001795207889792085

分享=0.0017536332336680529

代表=0.0017179545212076591

帮忙=0.0017146567512773445

国外=0.001696640911013477

面对=0.0016878488862844484

控制=0.001676324923501431

关系=0.0016623716525972247

数字=0.001638114554928359

留学生=0.001637580646353303

欧盟=0.001634473162330321

蔓延=0.0016136711737932228

包括=0.0016136460015196156

人民日报=0.0016129745255228554

防疫=0.0016029337593271604

过程=0.001599408737036144

非洲=0.0015920649489218968

改变=0.001569282162525834

暴发=0.001568864517664402

救治=0.0015529409068622624

预计=0.0015477564693921672

联合国=0.0015284576864524205

各界=0.0014683649469147556

主席=0.0014588575230995388

那时候=0.0014528610303550424

西方=0.0014481501508400738

专访=0.001414514186437946

空间=0.0013880045728853773

交流=0.0013835709602457128

戴多=0.0013817818435086073

topic 7 :

新冠=0.13635626746394655

肺炎=0.13621831406293783

武汉=0.06653798322282176

记者=0.013896496838279822

湖北省=0.013642512656865068

关注=0.01186286739721615

监狱=0.011463143819053563

感染=0.01137173716712999

清零=0.00976317274260385

湖北=0.009634023313178933

部门=0.008104207683858434

调查=0.008091845286129183

武汉市=0.007892437533110478

事件=0.007735807690739856

卫健委=0.00768087412365688

浙江=0.007675997427502396

山东=0.006964232532718742

李兰娟=0.005729015681294794

疫情=0.005446511425122439

持续=0.005317467943870374

人员=0.00524854292318894

发生=0.004905131662736265

系统=0.004842109206715294

关闭=0.004826423102081943

旅游=0.004390666169416469

人数=0.004374621869406848

时期=0.004359420318680049

展开=0.004290119054532729

接受=0.004239290871882123

释放=0.0041944570336854515

这位=0.004013175924572156

采访=0.0037232914503022214

母亲=0.0035485725467024843

急需=0.0034070018505643557

球员=0.0033485906112736734

相关=0.003190039561256176

解封=0.0031862509391385845

回应=0.00306088712199286

发布=0.0030202913862174097

确诊=0.0029428115002396453

获悉=0.0029137878576447978

浙江省=0.002804394074141604

联合调查组=0.002733526726345831

放松=0.0027110490366950477

二十=0.0027038721902889378

两周=0.0024756434206044484

共有=0.0024522029024096593

恳请=0.0024005451416551442

比赛=0.002388400018514619

伸出=0.0023613609031876994

澳大利亚=0.0023027130704093256

体育=0.0022239078181190393

隐瞒=0.00221586068430269

阶段=0.0021829343413213823

姑娘=0.0021765308173900258

姚晨=0.002163089994837854

援手=0.00215817197986003

查看=0.0021296055682256875

身患=0.0021153816077991725

杭州=0.0021094556691554013

白血病=0.002106300946763417

公布=0.00208421122249725

院士=0.002071844132815935

有望=0.0020484885012922445

重病=0.0019669740688464383

山东省=0.0019593578114712636

监狱管理局=0.001958430904636314

个位=0.0019493812187353612

呼救=0.001835694220900882

陷入绝境=0.0018125731235654476

第一=0.0018115283277026233

免职=0.0018004778212343151

训练=0.0017955518380758958

党委书记=0.0017649231903615165

NBA=0.0017582227015830276

骨髓移植=0.0017171436370333705

罪犯=0.0016891304069223416

任城=0.0016885952893380685

纳入=0.0016674078874050536

女子监狱=0.0016597121229338545

提醒=0.0016066790014772233

上班=0.0015932847398923422

网民=0.00155922806239171

全国=0.001516004833858706

成员=0.0014698220872973213

俱乐部=0.001436326951859418

省司法厅=0.0013896402092900876

情况=0.0013669556238023698

冲刺=0.001304933782566073

戈贝尔=0.0012873422408837436

刻意=0.0012758145482328943

球队=0.0012585930148598765

统计=0.0012520970477230167

两会=0.0012386984050968273

联盟=0.0012284543036027982

事实=0.001221570880849818

中新社=0.0012067907650775958

暂停=0.0011991612425383566

订正=0.0011786100719872277

核减=0.0011777414991939474

topic 8 :

肺炎=0.04203821230147933

医院=0.03851606966464839

湖北=0.036561318527023365

武汉=0.036552797334109766

医疗=0.03230801774811269

新冠=0.027353026559272277

医生=0.02678786018500481

感染=0.013915869743901927

医护人员=0.01324255165041597

去世=0.012554410080622701

支援=0.009602128727373238

李文亮=0.009534210070395715

武汉市=0.00832543097840479

护士=0.0077466824929889756

方舱=0.006096905622072181

记者=0.005809771169026917

收治=0.005742920380893227

上午=0.005403516301004049

病房=0.005214887755113907

救治=0.004925842830939064

妻子=0.004394253165743695

定点=0.004392201673513553

队员=0.004352159009054454

首批=0.004298919622031472

医护=0.004249429717524223

广西=0.004190328251781961

一线=0.00418884036099447

第一=0.003975150255620485

消息=0.003472538599025789

驰援=0.00342580846706375

防护服=0.003321563468395688

电影=0.0032278369565757626

人民医院=0.003013060566650606

抢救=0.002978373246486116

不幸=0.0029670175984361034

病人=0.002874370643829408

隔离=0.0027007116864376716

中心=0.002698121081740867

病逝=0.002597927074927772

发布=0.0025398445191435843

加油=0.002520325304128333

常凯=0.0025145691233844285

床位=0.0024947420285394654

患者=0.00238476354853906

院长=0.002377561621464797

武汉市中心医院=0.0023474212305389327

下午=0.0023270945913640255

此前=0.0023228236456996096

制片厂=0.00231668001747867

回家=0.0022843097886804382

孝感=0.002233665968301626

病区=0.0022092937050393296

银华=0.0022076038864397647

襄阳=0.0021865962493984015

最早=0.0021803706968159193

火神山=0.0021602564337871544

雷神山=0.002151313099148207

援助=0.002126662815268849

黄冈=0.0021053483000768726

婚礼=0.0019965995735971988

贵州=0.0019498212418684433

主任=0.0019452224849124285

江苏=0.0019308732894954173

出生=0.0019156363847578142

湖南=0.0018988395398364317

重庆=0.0018703302626326354

出征=0.0018619605473500058

同事=0.001839646032853199

离世=0.0017910145094569342

黄石市=0.0017517059763553309

训诫=0.00173734287042319

医学=0.0017222572330665902

无效=0.0017189883694767741

感谢=0.0016884823279203858

卫健委=0.0016714560840775808

黄冈市=0.0016665395743834847

小时=0.001666255615627365

紧急=0.0016487632519627965

平安=0.001647939384328024

武昌医院=0.001646890096366155

抗击=0.0016295670738318635

收到=0.0016267769281036837

黄石=0.0016161728161734222

孝感市=0.0016122044658501548

一路走好=0.001593509965852875

重症=0.0015903026991324763

资源=0.001586692261607055

抗疫=0.0015712906303553408

建成=0.0015540739887692464

凌晨=0.001536018998272997

医师=0.0015217368398411106

荆州市=0.0015141324706304457

该院=0.0014989605565347713

天堂=0.0014959762136441477

一家=0.0014340839317976773

对口=0.001400558856866482

抵达=0.001387610423725466

呼吸=0.0013609460183043244

中医院=0.0013583530156559523

福建=0.001351280232271965

topic 9 :

隔离=0.033519849748247676

人员=0.02913782836228745

北京=0.015236925346445414

接触=0.01310120664332472

社区=0.01087744669070581

小区=0.009686389292377173

发热=0.009598228004545873

患者=0.00772333775269722

消毒=0.007715547750524039

居民=0.007126766020656468

观察=0.007096084567379325

情况=0.006912091386546995

居家=0.006581475652021657

发现=0.006391938339328286

工作人员=0.006092849057763386

期间=0.005694671766694516

口罩=0.005555787830479398

聚集=0.00526746472458167

就诊=0.005256138607070357

市民=0.004772359910557264

活动=0.004562033864482102

员工=0.0045311539592693545

14天=0.004503603035243276

外出=0.00442497331538066

管理=0.004408336866524834

排查=0.0043402469329634425

男子=0.004307427841498462

广州=0.004281818387612541

西安=0.0041088800951260595

防护=0.004039003283287568

咳嗽=0.003990733520830882

检查=0.003981060591096965

北京市=0.0039725705506204224

疾控中心=0.0035257933727488896

郑州=0.003487551090118113

返回=0.003387812073716627

体温=0.003367578527708476

登记=0.0033356271147859675

相关=0.0032068343476725067

警方=0.0032065796132167866

健康=0.003072599458194351

女子=0.0030427336510884754

前往=0.003018087259198059

症状=0.002998535466769573

新发地=0.0029297847341713383

乘坐=0.0028526244608694977

门诊=0.0028429989154521934

封闭=0.002753468887837112

医学=0.00274703828192805

居住=0.0027334667963221644

成都=0.0027104803292170234

实行=0.002638672295390492

下午=0.002634010562794493

佩戴=0.0026238655477460017

超市=0.002568498369577698

隐瞒=0.002521680306078644

调查=0.0024924166748773596

场所=0.0024881273531461552

轨迹=0.0023103202416487408

上海=0.0022838601655577584

配合=0.0022610469441883825

酒店=0.002256653791010231

新京报=0.0021992676968718974

转运=0.0021901145483058685

车辆=0.0021310269480440563

区域=0.002108027414486926

自我=0.002105305671699932

快递=0.0020833953390125984

家中=0.0020802865418560897

深圳=0.001986849854290885

解除=0.001984558762575401

主动=0.0019111930477710532

街道=0.0019089910951815999

上班=0.0019017061335074616

上午=0.0019013356341709372

通报=0.0018498245762261834

发烧=0.0018465169653032224

陕西=0.0018283356610244263

证明=0.0018158996781467804

一律=0.0017631721246964315

列车=0.0017527375197979942

外卖=0.001747824633622236

记者=0.0017470092278537444

申报=0.0016583758433756518

通风=0.0016527436551129796

发病=0.001644043532945946

酒精=0.001619885540228211

防疫=0.00159732849096948

高铁=0.0015833453568029302

爆料=0.0015628380558598306

负责=0.0015582670079721983

电话=0.001557825315765816

聚餐=0.0015543541808658897

女儿=0.001554113893424432

信息=0.001518350414714974

消杀=0.001507126122707638

状态=0.0014612571285361716

疑似=0.0014476088031015934

单位=0.001437457230271844

一家=0.0014310765598288136

topic 10 :

江苏=0.012118256081997337

希望=0.012086905067143648

真的=0.011992109944924728

加油=0.009485910767433667

都是=0.007959039494192278

在家=0.00584040513798129

有人=0.005501440262696352

结束=0.0054989707826601135

生活=0.005331554058510326

孩子=0.0050095604738315674

出门=0.004513054523674359

不知道=0.0038766576627445658

网友=0.0038197544051422304

非典=0.0037422464623942425

感觉=0.0036749709943724685

担心=0.0034832602408507857

只能=0.003160707372634938

南京=0.003147113182954797

妈妈=0.0031423879125679842

老人=0.0030785786294250787

回来=0.0030696129816323446

害怕=0.003061834836450253

事情=0.003055314140200371

经历=0.003040837023499648

可怕=0.0028659068534393054

都在=0.0027186040412598765

家人=0.002654362751838722

明天=0.0025725870879956844

回家=0.002542060469861094

父母=0.0023978386905796943

不到=0.0023626114694096528

好好=0.0023569273545562285

朋友=0.0023356037621621853

地方=0.002308399348381089

春节=0.0022781158632664087

晚上=0.002238570851428107

早日=0.002216709386991685

特别=0.00218052479744729

想到=0.002165716319939158

儿子=0.0021636947750867993

宝宝=0.0021349125189208465

平安=0.0021237691756044847

战胜=0.0021230973134884327

日子=0.0021105840271076165

都会=0.0020453351655041964

城市=0.0020050699454256513

照顾=0.0019758098473679647

永远=0.0019752290365955784

家里=0.0019631260807963834

来了=0.0019161222619049464

太多=0.0019016083648167826

手机=0.0018621616756369416

喜欢=0.001847645958110596

终于=0.0018145316470715531

奶奶=0.0017738507222083578

在家里=0.0017685450417816227

就会=0.0017600445682949054

不好=0.0017064612676951504

日记=0.0016997313930169281

记录=0.0016755628085210628

赶紧=0.001657892431565097

确实=0.0016317450864591725

保护=0.0016313900778397636

不用=0.0015988242492905984

东西=0.0015884979100628492

身体=0.0015528885836618182

恐慌=0.0015461735505941212

快点=0.001544983256502743

回复=0.0015343153205719133

灾难=0.001532303217375132

老师=0.0015322396536133558

中国人=0.001524331220690191

昨天=0.0015079367825701316

本来=0.0014818697618059799

所有人=0.001457860490956369

科比=0.0014506413094881163

明白=0.001448601372677535

说话=0.0014319702636688733

几个=0.001423063489735607

天天=0.001417287140558914

世界=0.0014036829475222608

听到=0.0014018695232126915

女儿=0.0013987161147176082

春天=0.0013958997991209978

一个人=0.0013918385091347537

早上=0.001353860249210487

期待=0.001344099544079124

到来=0.0013439328685617966

理解=0.0013424265260595439

每个人=0.0013405651217130944

第一次=0.0013336459177904574

扬州=0.0013175458793606078

看着=0.0013102788290485833

选择=0.001309214973053274

情绪=0.001301371506822752

爷爷=0.0012833424185069724

度过=0.0012821504729116965

美好=0.0012713784968913194

自由=0.001250241389691847

早日康复=0.001243572759974441

topic 11 :

疫情=0.11782576506470258

防控=0.1039303691627224

工作=0.05218108943584725

指挥部=0.019332746662877973

新闻发布会=0.014126956888038899

会议=0.013721564043870423

应对=0.013018957076924177

落实=0.010306647089573434

中央=0.008782284788748233

发布=0.008461089612306473

风险=0.007930335559705967

推进=0.007812354686098663

部署=0.007656182464300697

介绍=0.007556891915036108

措施=0.007485546824054747

进一步=0.007466339149955841

工作领导小组=0.00744932016395413

重点=0.0071551115488676575

情况=0.0070518821625299155

肺炎=0.006951136940762204

联防=0.006535332234916714

联控=0.006513452087641012

国务院=0.006249971643178641

机制=0.0062486041051633716

社会=0.0061564924709275265

通知=0.005934292441202306

北京=0.005679181538799618

通告=0.005497048098173976

精神=0.005463277307476194

发展=0.004955511511028267

指导=0.004903206457229817

管理=0.004824516183825079

调整=0.004800529987107113

输入=0.004534308601514398

应急=0.0044933974831543

北京市=0.004347894531363806

贯彻=0.004321765597897734

统筹=0.00421851377967617

地区=0.004065184484373708

办公室=0.003810662626447921

下午=0.0037808212258840547

常态=0.0037600254030846643

单位=0.0037543033995424075

主持=0.003705165533371101

全市=0.0036548410028825473

响应=0.0036317410841148322

相关=0.0034184409842497095

人员=0.0034076258159071397

发布会=0.0033632118743753296

形势=0.0033573244475131356

经济=0.0032964352112154487

习近平=0.00311393834407441

公告=0.002951424213365622

河南=0.0028587315089818005

反弹=0.002646460417988497

讲话=0.002634822137278039

全省=0.002595702727409202

分区=0.0025065945789855353

管控=0.0024992403320152645

分级=0.0024290208729033295

印发=0.0024208014302538367

健康=0.0024089980332719437

党中央=0.0024073062181699883

河南省=0.0023917399641559755

决策=0.002293978565150649

总书记=0.0022923588102940137

成员=0.002253565083430154

我市=0.002224037434292611

综合=0.0021983187811472006

各项=0.002131187620523673

自治区=0.0020983720535788035

专题=0.0020384452250791977

人群=0.001983527081543513

市委=0.0019566932967263113

有序=0.0018991662821397212

指出=0.0018283230193199707

等级=0.0018147602897806928

卫生=0.0017466702562226252

机关=0.001726598968451966

领导小组=0.001705694005187893

二级=0.0016945505668400033

新闻发言人=0.0016763402825841

精准=0.001662414539730946

指示=0.001627385464493025

领导=0.0016160303837511427

婚姻登记=0.0016111169754649882

切实=0.0016028819973436185

实施=0.0015983882203456858

副主任=0.0015944804938369016

法治=0.001578187665913901

督导=0.0015526342653481174

分类=0.0015296613351602742

突发=0.0015237118240672696

完善=0.0014978519065653479

组长=0.0014757348801453312

处置=0.0014532469466494043

级别=0.0014253006043802206

内蒙古自治区=0.0014183911927085604

群众=0.0014074357928066396

企事业单位=0.0014033161063159991

topic 12 :

疫情=0.04035505027768628

健康=0.025780180158768345

肺炎=0.023638124177530206

发布=0.01925368314276975

新冠=0.01285346371023856

开学=0.012087715251674559

时间=0.011777514058630416

期间=0.010991351050114362

学生=0.010257531572608784

医务人员=0.008990537528957161

学校=0.008196422268280124

卫生=0.007886349105108355

措施=0.007443330808548273

网络=0.006635184522576433

高校=0.0064725382954403604

全国=0.005911846848577747

参加=0.005755566692404863

安排=0.005398388285462116

一线=0.00515632161457879

钟南山=0.004721528004768308

学习=0.004699298465605618

高考=0.004628391616641769

防疫=0.004591656855159654

情况=0.004584881535606633

教育=0.0044515923460613

指南=0.004366402077211412

组织=0.004306539157181603

五一=0.004263987990701087

广东=0.004165505429642097

推迟=0.0041396186736513425

公众=0.00412612529682515

机构=0.003986655336636979

现场=0.003661153353452115

线上=0.003655686031749049

提出=0.00359757287704274

教育部=0.003464786926227407

考试=0.0034238933633661617

公布=0.0033536384205684697

官方=0.00331577841719982

活动=0.0032795827406950184

幼儿园=0.0032713158796486

中小学=0.003271097993030355

峰值=0.0032397384261469136

依法=0.0031961478344271084

院士=0.0030461572912020966

专业=0.003043325908155203

网上=0.003038899859176537

提醒=0.0029804828308915483

方式=0.002953860063996794

培训=0.0029255024747108557

影响=0.0028715345005616917

复课=0.0028661058231839115

发生=0.0028629254188122727

案例=0.0028381254741895686

儿童=0.0028376217571075943

来了=0.002835004197411857

假期=0.0028285958125477805

返校=0.0028068517164721056

预计=0.002770804869894698

政府=0.0027700492723594823

指导=0.0027693488415718288

犯罪=0.0027504560485092856

原则=0.0027075763011631467

今日=0.0026972554709237947

中考=0.0026414514771265288

日前=0.002638286042429457

通知=0.0026222544861672905

知识=0.0025821021131614695

典型=0.0025768578394827824

子女=0.0025161480922853393

防护=0.0024642459528874143

在线=0.0024564912917890967

月底=0.002448023537412652

权威=0.00226465941387935

平台=0.002254444392991074

正式=0.0022530544395328884

下午=0.0022200949965741697

考生=0.0021780020326654585

增加=0.0021730814529122362

家长=0.0021573091192316966

师生=0.002156209217202379

建议=0.0021218955992013495

年级=0.00208971964547982

心理=0.0020746733177270486

开通=0.002055905310706939

毕业=0.00204335164210201

校园=0.0020287165209043525

附属=0.0020239946026112043

中下旬=0.0020185685244569966

提示=0.0019918704051559736

野生动物=0.001986167643601008

利用=0.0019594466878320067

停课=0.001955130369192848

法律=0.0019093687380603206

估计=0.0019038690029762136

小学=0.0018423481209676692

法院=0.0018376727527223415

内容=0.0018256115994519893

报名=0.0018178336580544018

工作=0.0017342326082271339

topic 13 :

疫情=0.07394539015295191

抗击=0.029461557822950207

抗疫=0.015193920722886417

防控=0.013627318961668376

一线=0.012712698775659222

全国=0.009418775479086207

工作=0.009083353636218226

防疫=0.008219528706063614

加油=0.007631160369808424

阻击=0.007071789572345865

生命=0.006945619478881647

万众一心=0.005806613021352054

致敬=0.005449128324795362

面对=0.005285757084978991

众志成城=0.0052652688412488345

力量=0.005045624132885617

发生=0.004805928519442567

青年=0.004672072012271651

打赢=0.004467059395517487

群众=0.004094062147580094

坚守=0.003906385311735112

志愿者=0.00390106804151297

担当=0.00365776798641064

爱心=0.0035580581871749156

宣传=0.0035565822062206907

第一线=0.0035540242441423836

奋战=0.003545568979243607

社区=0.003545560477632945

主动=0.003426855923524817

始终=0.0034161152753002743

党员=0.0034010400947249446

前线=0.003201569431006478

责任=0.0030687862354972966

英雄=0.0030625512092002758

捐款=0.002995247557779747

努力=0.0029951937511090696

民警=0.002916050528348982

最美=0.002911420277279102

全民=0.002898578377221134

贡献=0.002807165228703435

领导=0.0027968327849599538

守护=0.0026967610949689053

干部=0.0026391321777452046

青春=0.002620115383344193

活动=0.002579249679871605

时期=0.0025709014205959483

参与=0.002569040799266993

文明=0.002519369075670683

基层=0.002518701608215883

胜利=0.0025041566426437247

脱贫=0.0024960556669050052

突如其来=0.0024516508909345194

岗位=0.002376358956184201

攻坚=0.0023483999118692274

平安=0.002341966022356994

在一起=0.0023367058401835743

公安=0.0022450513198722572

组织=0.002166607068679734

同胞=0.0020587928890101535

号召=0.0020186704229789394

逆行=0.001996055635628039

斗争=0.001975909285263764

牺牲=0.0019206324706656066

社会=0.001886135244939764

响应=0.0018377022579070135

关键=0.0018246072983788629

故事=0.001807592158767533

志愿=0.0017992810676405373

战士=0.0017725298312468547

医护人员=0.0017061876104748788

精神=0.0016909500054420373

警察=0.001655165878881584

慰问=0.001653260915447219

发挥=0.001649490278200836

城市=0.0016493743643439555

兰州=0.0016130789909246043

逆行者=0.0016128840120371592

感谢=0.001570741764470942

医务工作者=0.0015663308576994092

第一时间=0.0015524878357480218

战胜=0.0015018033068287388

严峻=0.0014956928808991486

身体健康=0.001479175260143214

作用=0.0014717537040204118

信心=0.0014656635675523912

牵动=0.0014554322137027558

战场=0.0014460975133147112

在前=0.0014407063183771215

打响=0.0014093862855874097

倡议=0.0013976034082356318

白衣天使=0.0013673445270186504

坚定=0.0013627035735526778

江西=0.0013313460430566411

烈士=0.0013294357049633047

冲锋=0.0013259273365090493

温暖=0.0013229061980581844

安徽=0.0013227773187440921

奉献=0.001321182224965333

使命=0.0013163288444937886

防线=0.001301790973892186

topic 14 :

美国=0.09520337892817088

新冠=0.07302803458117231

死亡=0.03228990459199922

全球=0.029626742452011547

时间=0.028612013973858513

肺炎=0.019701576343670472

特朗普=0.019238542064196885

人数=0.015876937646682392

世卫组织=0.014173394840753632

确诊=0.014012417129199133

超过=0.013486698824369939

数据=0.01193908042126944

英国=0.01058520657110437

意大利=0.010176458717322137

显示=0.009183617614195382

国家=0.007242850364638132

印度=0.0069566792619814586

巴西=0.006454129166929002

小时=0.0064383832572709404

实时=0.006179430808119698

霍普金斯大学=0.006129749779687172

法国=0.005765346603257012

欧洲=0.005649304303475894

约翰斯=0.005608511044837244

报道=0.005287830807079218

单日=0.005032176481910856

北京时间=0.004732984705083828

已有=0.004556240180216515

统计=0.0045377953776479085

病毒感染=0.004434344978434545

西班牙=0.004081444134750868

纽约州=0.00394981787603631

白宫=0.003897986407491128

德国=0.003890580736891917

地区=0.00386804445957346

民众=0.0038027982780324296

系统=0.0037740651426174934

媒体=0.00371715352535323

总干事=0.003562069555647585

总统=0.0032575370854355675

谭德塞=0.0032172668838406893

纽约=0.0031718492512070495

流行=0.003116579633559491

10万=0.0030126179901720563

统计数据=0.0029935486321247258

世界卫生组织=0.002860133365208695

全美=0.0027710957626114804

发布=0.002767496977719232

美国人=0.002715817052335174

警告=0.0027126757658773853

至少=0.0026892195051745633

州长=0.0026496662647387563

美国总统=0.002588640518890252

世卫=0.0024287061573379006

东部=0.0023334867344847114

当天=0.0022843349771253956

市长=0.0022524412813919167

暴发=0.0022157526329016556

突破=0.002153902526323465

数字=0.0021431776702690144

官员=0.0020777920621523325

最新=0.0020702808708461047

去年=0.00206726645664016

抗议=0.002033103428407787

发布会=0.0020019558538640365

最多=0.001982180582613498

疫情=0.001908988782128596

增加=0.0018556414179694417

紧急=0.001843167948984494

蔓延=0.001792516101394209

升至=0.0017813723418114043

福奇=0.0017705957335763076

包括=0.0017315275695973735

世界=0.0017032194428515815

社交=0.00161425764659636

该国=0.0015637922598416552

下午=0.001508040410981473

相比=0.001504675654141923

科莫=0.0014513911787491535

此前=0.0014215829986950358

数量=0.0014076394380560014

公布=0.0014067276662110564

每日=0.0013969401865699985

约翰=0.0013512551379734315

新闻网=0.0013395087337791265

承认=0.0013354904831572396

封锁=0.0013129358210408066

死亡率=0.001296745896995016

百万=0.0012964110365881834

死于=0.0012715627251083444

更新=0.001233891074762902

成就=0.0012065934948261793

死者=0.0012014898659771798

例行=0.00119949020625002

旅行=0.0011842100537146857

人民日报=0.001184120971649304

卫生=0.0011783409208447257

加拿大=0.0011684917198802277

CNN=0.00114785489501145

美国政府=0.001138074318039628

topic 15 :

肺炎=0.10797117942012349

新冠=0.09852528935264902

视频=0.0774131951151668

新闻=0.052050669422467354

微博=0.04930901840681995

央视=0.025854740106087834

信息=0.020111423277060593

求助=0.01434812908550532

关注=0.012340379146523141

转发=0.010878852644030495

提供=0.009817250486895291

病人=0.00853860279879614

危重=0.008171896953022261

农业=0.00788833650474851

扩散=0.007559316228771461

通道=0.0073038286045890195

直播=0.006695312904475841

平台=0.006191010795526393

全国=0.006052239020751881

分享=0.005917685125240303

人民日报=0.005282573653473971

新浪=0.004996586742446826

地区=0.0048167810780256936

客户端=0.004560600416212076

腾讯=0.00448139650470979

凤凰=0.0042669928981080366

联合=0.003934062613271461

转给=0.003930851726230164

发送=0.00390660691022891

解决=0.003904178869384231

尽力=0.0036653479180537565

政府=0.0035292732583448293

病患=0.003497902774990935

格式=0.003452577808895034

接收=0.0032994196686349313

免费=0.0032138769897064267

疾病=0.003162643028411922

相关=0.0030829567769066818

地图=0.003055171178811005

粮食=0.002847022841521855

好消息=0.002702543985629011

中新=0.00270174835618126

澎湃=0.002672681929989077

网易=0.002557227392909395

来了=0.0025505111447655967

农村=0.0023937700797181318

部长=0.0022339888312335396

战役=0.0022051100543426356

保障=0.002124572142194932

开启=0.0020796193044695286

更新=0.0020235363250210455

急需=0.0020087739456961045

停止=0.0019977335798471883

人民网=0.0019931443369661608

点击=0.0019006281369690298

有望=0.0018976561255033262

来源=0.0018763640011430858

征集=0.0018503393284778737

微信=0.0018237782398877348

生命=0.0017935416975599441

商户=0.0017763949961596452

朋友=0.0017660703128663745

核实=0.001712542778761192

泸州=0.001708210754091359

紧急=0.0016298334373627558

via=0.0015881179081451903

同行=0.0015584076916901216

视点=0.0015535321142263769

旗下=0.001490031881016746

暴发=0.001453168759765094

能力=0.001424196924414736

热点=0.0013968193839013071

小微=0.0013935257476480809

丰收=0.0013933376532451695

最新=0.0013758328881163836

大会=0.0013504522403254007

新华=0.0013374660106540493

央视网=0.001323045718347302

总台=0.0013136098778812003

分开=0.0013058895362236108

身边=0.0012773216194683437

关键=0.0012518212654107994

店主=0.0012430401896253288

检验=0.001225777028839826

农产品=0.0012202464078212103

给予=0.0012199673554382456

头条=0.0012023439223201014

依然=0.0011620411356658992

转交=0.0011596923718662957

癌症=0.001157203614817127

终于=0.0011312402256201902

#O=0.0011211516775596973

讲述=0.0011016639527814476

信心=0.0011012000327483955

即日=0.0011000259388844833

谢谢=0.0010904624029478437

回应=0.0010818952695146754

热门=0.00106534172857868

看点=0.0010617334239957418

公益=0.0010555848153486072

topic 16 :

肺炎=0.09403569900168375

新冠=0.07294900911305251

新型=0.06994309946231103

冠状病毒=0.06249978098441443

检测=0.05798138711428727

感染=0.04714277310144408

病毒=0.0385310561986929

伊朗=0.026900075431103024

核酸=0.025685335384851054

阳性=0.01760115416054727

报道=0.013462779618700009

原因=0.009993815891774092

副总统=0.008105687201546651

不明=0.007961969947360986

消息=0.007101400634049168

媒体=0.006835512936776513

阴性=0.006737319574418906

官方=0.005412543303290916

大使=0.004594471797543164

华南海鲜市场=0.0045870084452239316

接受=0.004307244121883715

病原体=0.004112922020266731

刚刚=0.003913826992027577

确认=0.003908505667452027

家庭=0.003885994471269854

去世=0.003787553939147001

梵蒂冈=0.003778501023397953

初步=0.003578971392125213

负责=0.003518434373978722

快讯=0.0034297538899220885

事务=0.00280693287548467

国家=0.0025681091208682987

样本=0.002497772414870821

沙希=0.002378526681694643

霍斯=0.0023479599671909477

通讯社=0.0023048427465373954

检出=0.0023039982425099325

专家=0.0022762266405152885

卫生部=0.0022629973808093807

电视台=0.002241810447745266

病原=0.00220817297872878

测试=0.0021827675199137036

进展=0.002181012968588544

苏梅=0.0021784232729918176

卫生=0.002058504348769609

此前=0.002055777235635457

第一=0.002054416362951553

死于=0.0019636939015024164

周四=0.0019132195173067982

担任=0.0019125026812964543

实验室=0.0018903972383073626

记者=0.0018863073797436012

海鲜=0.0018403935740372052

彭斯=0.0018391292377140404

判定=0.0017821800955368412

方法=0.0017426464863600308

华南=0.0016931059215081615

哈萨克斯坦=0.0016690637544317108

国有=0.0016509222508362684

妇女=0.0016393704639918985

上午=0.001638127541671668

副部长=0.0016345369696837654

高官=0.0016297897506700144

革命=0.0015762108096654967

筛查=0.0015077915555762502

本次=0.001455992279246244

引发=0.0014416585115428068

伊斯兰共和国=0.0014028304888822577

基因组=0.0013970254575682527

发现=0.0013818039795661823

试剂盒=0.001379223628516152

阿拉伯=0.0013741061660191814

库姆=0.0013336112720329623

女性=0.0013050226646818747

伊斯兰=0.0012807825921500805

咽拭子=0.0012741060663576445

环球网=0.0012264286214526863

序列=0.0012140659746675553

教士=0.0011947346885666957

哈迪=0.0011943605049806111

起源=0.0011937033895467748

病毒感染=0.0011915098454749634

IRNA=0.0011728790356453991

采样=0.001159917016049794

评估=0.0011590224453757777

老板=0.0011501012265428968

Hadi=0.0011474956977660618

Khosroshahi=0.0011198954857628382

中东=0.0010936372440951186

鉴定=0.0010845819300502088

Ebtekar=0.0010813348598358837

Masoumeh=0.0010806492526218828

话题=0.0010403149651494312

刊文=0.0010210749764490471

外媒=0.001007397235622294

Al=9.97566505255976E-4

Arabiya=9.94444206214134E-4

《科学》=9.940098095198838E-4

全员=9.81792740986824E-4

杂志=9.406855978005782E-4
